# FPN1–8C>G Polymorphism as a potential genetic modifier of iron overload in transfusion-dependent β-Thalassemia major patients receiving HbF augmentation therapy

**DOI:** 10.12669/pjms.42.9.15478

**Published:** 2026-09

**Authors:** Maeesa Wadood, Muhammad Younus Jamal Siddiqi, Iram Nazir, Muhammad Rizwan

**Affiliations:** 1 Maeesa Wadood (MBBS, M.Phil., MS) Executive Medical Director, Muhammadi Hematology, Oncology Services & Welfare Foundation, Karachi, Pakistan. Professor, Department of Pathology, Baqai Medical University, Karachi, Pakistan; 2 Muhammad Younus Jamal Siddiqi (MBBS, Ph.D.) Associate Professor, Dow University of Health Sciences, Karachi, Pakistan. Research Supervisor and Assistant Professor (Adjunct), Baqai Institute of Hematology, Baqai Medical University, Karachi, Pakistan; 3 Iram Nazir (MBBS, M.Phil.), Baqai Institute of Hematology, Baqai Medical University, Karachi, Pakistan; 4 Muhammad Rizwan (MBBS, M.Phil., Ph.D.) Deputy Director and Associate Professor, Baqai Institute of Hematology, Baqai Medical University, Karachi, Pakistan

**Keywords:** β-Thalassemia, Ferroportin, Genetic, Hydroxyurea, Iron Overload, Polymorphism

## Abstract

**Objectives:**

To assess the association of the FPN1–8C>G polymorphism with iron overload and iron-regulatory biomarkers in transfusion-dependent β-thalassemia major patients receiving fetal hemoglobin (HbF) augmentation therapy.

**Methodology:**

This cross-sectional study was conducted from January 2024 to May 2025 at Fatima Hospital, Baqai Medical University, and Muhammadi Thalassemia Centre, Karachi, Pakistan. A total of 120 transfusion-dependent β-thalassemia major patients receiving hydroxyurea-based HbF augmentation therapy for at least six months were recruited through consecutive sampling. Conventional iron indices and serum hepcidin, erythroferrone (ERFE), growth differentiation factor-15 (GDF-15), and soluble transferrin receptor (sTfR) were measured. PCR-RFLP was performed for FPN1–8C>G genotyping, with Sanger sequencing confirmation.

**Results:**

The FPN1–8C>G variant was present in 39 (32.5%) patients. Iron overload was significantly associated with genotype (*p*=0.012). Variant carriers (CG+GG) had higher odds of iron overload than CC carriers (OR=5.67; 95% CI: 1.60–20.14; *p*=0.003), and the association remained significant after adjustment for iron-chelation regimen and duration of transfusion exposure (aOR=5.26; 95% CI: 1.43–19.31; *p*=0.012). Serum iron, ferritin, transferrin saturation, ERFE, and GDF-15 increased across CC, CG, and GG genotypes, whereas TIBC, UIBC, hepcidin, and sTfR decreased (all *p*<0.001).

**Conclusions:**

FPN1–8C>G was significantly associated with greater iron burden and altered iron-regulatory biomarkers. It may represent a potential genetic marker of susceptibility to iron overload in transfusion-dependent β-thalassemia major; however, prospective validation is required.

## INTRODUCTION

In Pakistan, β-thalassemia remains a substantial public health burden, with a reported carrier frequency of 5–8%.^1^,^2^. Although advances in supportive care and transfusion protocols have improved survival, repeated blood transfusions result in progressive iron accumulation.^3^ Excess iron deposition in the liver, heart, and endocrine organs remains an important cause of long-term morbidity and mortality in transfusion-dependent patients.^4^ Thus, understanding factors that contribute to inter-individual variability in iron burden is clinically significant.^5^

Systemic iron homeostasis is primarily regulated by the hepcidin–ferroportin axis, a central pathway involved in iron metabolism.^6^ Ferroportin, encoded by the *SLC40A1* gene, is the principal cellular iron exporter and mediates iron release from enterocytes, macrophages, and hepatocytes into the circulation.^7^ Hepcidin, a hepatic peptide, maintains iron balance by binding to ferroportin and promoting its internalization and degradation. Disruption of this pathway may contribute to abnormal iron accumulation.^8^

The FPN1–8C>G polymorphism has been investigated as a potential genetic factor contributing to variability in iron homeostasis.^9^ However, clinical evidence remains limited. El-Gharbawi et al. reported an association between FPN1–8C>G and higher liver iron concentration in Egyptian patients with β-thalassemia major, although no significant association with serum ferritin was observed.^10^ Other *SLC40A1* variants have also been associated with variability in iron loading and related phenotypes.^11^ Nevertheless, evidence regarding FPN1–8C>G in populations outside Egypt, particularly in South Asia, remains scarce.

Iron homeostasis in β-thalassemia is also influenced by erythropoietic and iron-regulatory mediators, including erythroferrone (ERFE), growth differentiation factor-15 (GDF-15), hepcidin, and soluble transferrin receptor (sTfR).^12^,^13^ These biomarkers provide additional insight into erythropoietic activity and iron-regulatory signaling beyond conventional indices such as serum ferritin and transferrin saturation.^14^,^15^

Hydroxyurea-induced fetal hemoglobin (HbF) augmentation, an adjunctive therapy in β-TM patients, may reduce transfusion requirements in responsive patients, potentially reducing transfusional iron burden.^16^ However, substantial variability in iron burden may persist among patients receiving transfusion support, iron chelation, and HbF augmentation therapy.^17^ The contribution of FPN1–8C>G to this variability remains uncertain, particularly in South Asian populations, and its relationship with iron-regulatory biomarkers has not been adequately characterized. To our knowledge, this is the first study from Pakistan to evaluate the FPN1–8C>G polymorphism in relation to conventional iron indices and multiple iron-regulatory biomarkers in transfusion-dependent β-thalassemia major patients receiving HbF augmentation therapy. Therefore, this study aimed to assess the association of the FPN1–8C>G polymorphism with iron overload and iron-regulatory biomarkers in this population.

## METHODOLOGY

This cross-sectional study was conducted from January 2024 to May 2025 at Fatima Hospital, Baqai Medical University, and the Muhammadi Thalassemia Center, Karachi, Pakistan.

### Ethical approval:

The study protocol was reviewed and approved by the Institutional Review Ethics Board (IREB) of Baqai Medical University (Approval No. BMU-IREB/05-2023, dated 20 November 2023). At the time of enrollment, written informed consent was obtained from all participants or their legal guardians. Participant anonymity and voluntary participation were maintained throughout the study. All study procedures were conducted in accordance with the principles outlined in the Declaration of Helsinki and institutional ethical requirements.

### Study population, Sampling technique, and Sample size:

The study population comprised patients with transfusion-dependent β-thalassemia major recruited through consecutive sampling from the participating centers. Based on a reported 34% frequency of the heterozygous FPN1–8C>G genotype in the study by El-Gharbawi et al., the sample size was calculated using a single-population proportion formula with 95% confidence and 8.5% absolute precision, yielding a required sample of 120 participants.^10^

### Inclusion criteria:

- For this study, diagnosed cases of β-thalassemia major, confirmed by high-performance liquid chromatography (HPLC), hemoglobin electrophoresis, and/or molecular genetic testing, were recruited in accordance with institutional standards. Eligible participants were transfusion-dependent patients aged two to thirty years who had received more than ten units of red cell transfusions and had been receiving HbF augmentation therapy with oral hydroxyurea at 10-mg/kg/day for at least six months. Response to hydroxyurea was assessed according to changes in packed red-cell transfusion requirements. Patients who became transfusion-independent were classified as good responders; those with >50% reduction in transfusion requirement as partial responders; and those with <50% reduction as non-responders, as described by Ansari et al.^18^

### Exclusion criteria:

- Patients with β-thalassemia minor or other hemoglobinopathies, acute infections, chronic liver or renal disease unrelated to thalassemia, pregnancy, or documented non-compliance with prescribed iron chelation therapy were excluded. Among patients for whom iron chelation therapy had been prescribed, non-compliance was operationally defined as intake of <70% of prescribed doses during the preceding six months, based on medical records and patient or caregiver reporting. Patients in whom iron chelation had not been prescribed according to the institutional treatment protocol and whose serum ferritin remained <1000 ng/mL were not classified as non-compliant**.**

### Clinical assessment and Data collection:

Data were collected using a structured research proforma documenting sociodemographic characteristics, clinical history, transfusion exposure, HbF augmentation therapy, iron chelation regimen and duration, and relevant clinical findings. Iron chelation was categorized as monotherapy with deferasirox, dual therapy with deferiprone plus deferoxamine, or triple therapy with deferasirox, deferiprone, and deferoxamine. Patients not receiving chelation because they had not met the predefined criteria for prescribing iron chelation therapy were recorded separately and were not considered non-compliant.^19^ The duration of transfusion exposure and iron-chelation regimen were recorded as potential confounding variables and subsequently evaluated in multivariable analysis.

### Laboratory investigations:

Under aseptic conditions, 8-mL of venous blood was collected from each participant; 5-mL was transferred to a serum separator tube for biochemical and immunoassay analyses, and 3 mL to an EDTA tube for molecular testing. Serum iron and unsaturated iron-binding capacity (UIBC) were measured using the Cobas C 111 analyzer (Roche Diagnostics, Mannheim, Germany). Total iron-binding capacity (TIBC) was calculated as serum iron + UIBC, and transferrin saturation (%) as [serum iron/TIBC] × 100. Serum ferritin was measured by electrochemiluminescence immunoassay (ECLIA) on the Cobas e 411 analyzer (Roche Diagnostics, Mannheim, Germany).

Serum hepcidin, erythroferrone (ERFE), growth differentiation factor-15 (GDF-15), and soluble transferrin receptor (sTfR) were quantified by commercially available enzyme-linked immunosorbent assay (ELISA) kits according to the manufacturers’ instructions. Hepcidin and GDF-15 were measured using Quantikine™ ELISA kits (R&D Systems, Minneapolis, MN, USA), ERFE using ELISA kits from ELK Biotechnology Co., Ltd. (Sugar Land, TX, USA), and sTfR using a human sTfR ELISA kit from Cusabio Biotech Co., Ltd. (Wuhan, China).

### Operational definition of iron overload:

Serum ferritin was used as an operational measure of iron burden. Based on the serum ferritin strata described by Taher and Saliba for transfusion-dependent thalassemia, 1500 ng/mL was selected as the prespecified cutoff for the present analysis. Patients were therefore dichotomized into serum ferritin <1500 ng/mL and ≥1500 ng/mL groups.^20^

### Genetic analysis:

Genomic DNA was extracted from peripheral blood leukocytes using the QIAamp DNA Blood Mini Kit (Qiagen GmbH, Hilden, Germany), and DNA concentration and purity were assessed using a NanoDrop 2000 spectrophotometer (Thermo Fisher Scientific, USA). The FPN1−8C>G polymorphism was analyzed using polymerase chain reaction–restriction fragment length polymorphism (PCR-RFLP). PCR amplification was performed on the Montania Real-Time PCR System (Anatolia Geneworks, Istanbul, Türkiye) using the forward primer 5′-CCAGTTCCTTGCACTCCTG-3′ and reverse primer 5′-CATCCTCTCTGGCGGTTG-3′. Each reaction was prepared in a final volume of 25-µL containing PCR Master Mix, forward and reverse primers, template DNA, and nuclease-free water. Amplification consisted of initial denaturation at 95°C for five minutes, followed by 30 cycles of denaturation at 95°C for 30 seconds, annealing at 60°C for 30 seconds, and extension at 72°C for 60 seconds, with a final extension at 72°C for five minutes. The amplified 129-bp PCR product was subjected to restriction digestion with BstUI (New England Biolabs, Inc., USA). Digested products were separated on a 3% high-resolution agarose gel to facilitate discrimination of the expected small DNA fragments. The wild-type CC genotype yielded an undigested 129-bp fragment, the heterozygous CG genotype yielded 129-, 85-, and 44-bp fragments, and the homozygous variant GG genotype yielded 85- and 44-bp fragments. All detected variant genotypes were subsequently confirmed by Sanger sequencing.

### Statistical analysis:

Data were tabulated in Microsoft Excel and analyzed using SPSS version 27.0 (IBM Corp., Armonk, NY, USA). Continuous variables were summarized as mean ± SD with 95% CIs for descriptive purposes, and categorical variables as frequencies and percentages. Normality was assessed using the Shapiro–Wilk test, and inferential tests were selected according to the data distribution and homogeneity of variance. Welch’s ANOVA was used for serum iron, TIBC, UIBC, and transferrin saturation; one-way ANOVA for hepcidin; and the Kruskal–Wallis test for serum ferritin, ERFE, GDF-15, and sTfR.

Genotype frequencies were expressed as n (%), allele frequencies were calculated from observed genotype counts, and Hardy–Weinberg equilibrium was assessed using an exact test. The association between the three FPN1–8C>G genotypes and iron-overload status was evaluated using the Fisher–Freeman–Halton exact test. For carrier analysis, CG and GG genotypes were combined and compared with the CC genotype, and odds ratios (ORs) with 95% CIs were calculated. The association between FPN1–8C>G carrier status and iron overload was further assessed using multivariable binary logistic regression, adjusting for iron-chelation regimen and duration of transfusion exposure. Spearman’s rank correlation was used to assess associations between genotype and iron-regulatory biomarkers, with genotype coded ordinally as CC=1, CG=2, and GG=3, and 95% CIs estimated by non-parametric bootstrapping. No data were missing for variables included in the primary analyses. A two-sided *p*-value <0.05 was considered statistically significant.

## RESULTS

### Sociodemographic and Clinical characteristics:

A total of 120 patients with transfusion-dependent β-thalassemia major were included, with a mean age of 9.07 ± 4.60 years; 59.2% were male. Parental consanguinity was reported in 78.3%, and 54.2% had a family history of thalassemia. The mean duration of transfusion exposure was 7.87 ± 4.45 years. Chelation regimens and responses to HbF augmentation varied across the cohort, with non-responders constituting the largest response category (53.3%) (Table-I). Fourteen patients (11.7%) were not receiving chelation because they had not met the predefined criteria for initiation of therapy and were therefore not considered non-compliant.

**Table-I:** Sociodemographic characteristics of Beta Thalassemia major patients.

***Age in years***	
Mean Range	9.07 ± 4.60 2 - 24
***Gender***	
Male	71 (59.2%)
Female	49 (40.8%)
***Parental Consanguinity***	
Yes	94 (78.3%)
No	26 (21.7%)
***Family History of Thalassemia***	
Yes	65 (54.2%)
No	55 (45.8%)
***Transfusion years***	
Mean	7.87 ± 4.45
Range	1.5 – 23.5
***Iron chelation therapy***	
Deferasirox	40 (33.3%)
Combined therapy	44 (36.7%)
Triple therapy	22 (18.3%)
No iron chelation therapy	14 (11.7%)
***Hb F Augmentation Therapy Response***	
Good responders	25 (20.8%)
Partial responders	31 (25.8%)
Non-responders	64 (53.3%)

Data are presented as n (%) or mean ± SD (range), as appropriate. HbF = fetal hemoglobin.

### FPN1–8C>G Genotype and Iron overload:

The CC, CG, and GG genotypes accounted for 67.5%, 28.3%, and 4.2% of patients, respectively, with a G-allele frequency of 18.3%. The genotype distribution was consistent with Hardy–Weinberg equilibrium (exact *p*=0.544). Serum ferritin-defined iron overload (≥1500 ng/mL) was significantly associated with the FPN1–8C>G genotype (*p*=0.012), with the proportion of patients with iron overload increasing across CC, CG, and GG genotypes. Variant carriers (CG+GG) had significantly greater odds of iron overload than CC carriers (OR=5.67, 95% CI 1.60–20.14; *p*=0.003). The association remained significant after adjustment for iron-chelation regimen and duration of transfusion exposure (aOR=5.26, 95% CI 1.43–19.31; *p*=0.012) (Table-II).

**Table-II:** Association of FPN1–8C>G Genotypes with Serum Ferritin–Defined Iron Overload.

Genotypes	Serum Ferritin <1500 ng/mL	Serum Ferritin ≥1500 ng/mL	p-value
CC (Wild type)	26 (32.1%)	55 (67.9%)	0.012
CG (Heterozygous Variant)	3 (8.8%)	31 (91.2%)	
GG (Homozygous Variant)	0 (0.0%)	5 (100%)	
Total	29 (24.2%)	91 (75.8%)	
***Allele Frequency and Hardy–Weinberg Equilibrium Analysis***			
*C allele* *n (%)*	*G Allele* *n (%)*	*Observed/ Expected Genotypes*	*Exact HWE* *p-value*
196 (81.7%)	44 (18.3%)	CC= 81/80.03 CG=34/35.93 GG =5/4.03	0.544
***FPN1–8C>G Carrier analysis***			
Genotype Comparison	OR	95% CI	p-value
CG + GG vs CC (Unadjusted)	5.67	1.60 - 20.14	0.003
CG + GG vs CC (Adjusted)*	5.26	1.43–19.31	0.012

Data are presented as n (%). Percentages for iron-overload categories were calculated within each genotype. The overall association between FPN1–8C>G genotypes and iron-overload status was assessed using the Fisher–Freeman–Halton exact test. Allele frequencies were calculated from 240 alleles; observed/expected genotype counts are shown for CC, CG, and GG, respectively, and the genotype distribution did not significantly deviate from Hardy–Weinberg equilibrium (exact p=0.544). Carrier analysis compared CG+GG with CC using Fisher’s exact test. The adjusted OR was estimated using multivariable binary logistic regression controlling for iron-chelation regimen and duration of transfusion exposure. OR = odds ratio; CI = confidence interval; HWE = Hardy–Weinberg equilibrium. A p-value <0.05 was considered statistically significant.

### Iron-regulatory biomarkers across FPN1–8C>G Genotypes:

Significant genotype-related differences were observed across the evaluated iron-regulatory biomarkers. Serum iron, ferritin, transferrin saturation, ERFE, and GDF-15 increased progressively across CC, CG, and GG genotypes, whereas TIBC, UIBC, hepcidin, and sTfR decreased (all *p* < 0.001) (Table-III).

**Table-III:** Comparative analysis of Iron Regulatory Biomarkers among FPN1–8C>G Genotypes

Biomarkers	CC n = 81	CG n= 34	GG n= 5	Test Statistics	p-value
Serum Iron (µg/dL)	203.12 ± 14.07 (200.01 – 206.23)	213.46 ± 10.58 (209.77 – 217.15)	222.91 ± 6.60 (214.72 – 231.11)	Welch F = 20.01	<0.001
TIBC (µg/dL)	323.44 ± 7.04 (321.88 – 324.99)	318.27 ± 5.29 (316.43 – 320.12)	313.54 ± 3.30 (309.44 – 317.64)	Welch F = 20.01	<0.001
UIBC (µg/dL)	120.32 ± 21.11 (115.65 – 124.99)	104.82 ± 15.87 (99.28 – 110.35)	90.63 ± 9.90 (78.33 – 102.93)	Welch F = 20.01	<0.001
Serum Ferritin (ng/mL)	2643.59 ± 1717.28 (2263.87 – 3023.31)	4007.82 ± 1908.38 (3341.96 – 4673.69)	5914.00 ± 1680.01 (3827.99 – 8000.01)	H = 20.07	<0.001
Transferrin Saturation (%)	62.92 ± 5.69 (61.66 – 64.18)	67.14 ± 4.40 (65.60 – 68.68)	71.12 ± 2.83 (67.60 – 74.63)	Welch F = 19.54	<0.001
Hepcidin (ng/mL)	33.70 ± 9.36 (31.63 – 35.77)	24.72 ± 8.67 (21.69 – 27.74)	17.74 ± 6.92 (9.14 – 26.34)	F = 16.82	<0.001
ERFE (ng/mL)	4.75 ± 2.16 (4.28 – 5.23)	6.49 ± 2.19 (5.72 – 7.28)	8.67 ± 1.83 (6.40 – 10.93)	H = 20.70	<0.001
GDF-15 (pg/mL)	792.84 ± 510.62 (679.93 – 905.75)	1186.30 ± 553.25 (993.26 – 1379.34)	1777.37 ± 502.46 (1153.48 – 2401.26)	H = 19.68	<0.001
sTfR (ng/mL)	2142.18 ± 667.81 (1994.51 – 2289.84)	1613.91 ± 616.08 (1398.95 – 1828.87)	1039.99 ± 425.47 (511.71 – 1568.28)	H = 20.67	<0.001

Data are presented uniformly as mean ± SD (95% CI) for descriptive consistency. The choice of inferential test was based on normality and homogeneity of variance. Welch’s ANOVA was used for serum iron, TIBC, UIBC, and transferrin saturation; one-way ANOVA for hepcidin; and the Kruskal–Wallis test for serum ferritin, ERFE, GDF-15, and sTfR. TIBC = total iron-binding capacity; UIBC = unsaturated iron-binding capacity; ERFE = erythroferrone; GDF-15 = growth differentiation factor-15; sTfR = soluble transferrin receptor; SD = standard deviation; CI = confidence interval; F = analysis-of-variance statistic; H = Kruskal–Wallis statistic. p<0.05 was considered statistically significant.

### Correlation with FPN1–8C>G Genotype:

Spearman correlation analysis supported these genotype-related trends. FPN1–8C>G genotype was positively correlated with serum iron, ferritin, transferrin saturation, ERFE, and GDF-15 and inversely correlated with TIBC, UIBC, hepcidin, and sTfR (all *p* < 0.001) (Table-IV). The strongest correlation was observed with serum hepcidin (ρ = −0.465).

**Table-IV:** Correlation of FPN1–8C>G Genotype with Iron Regulatory Biomarkers

Biomarkers	Spearman ρ	95% CI*	p-value
Serum Iron	0.399	(0.240 to 0.541)	<0.001
TIBC	-0.398	(-0.541 to -0.238)	<0.001
UIBC	-0.398	(-0.542 to -0.235)	<0.001
Serum Ferritin	0.399	(0.237 to 0.541)	<0.001
% Transferrin Saturation	0.399	(0.237 to 0.542)	<0.001
Serum Hepcidin	-0.465	(-0.598 to -0.315)	<0.001
Erythroferrone	0.405	(0.241 to 0.550)	<0.001
GDF-15	0.394	(0.233 to 0.535)	<0.001
sTfR	-0.405	(-0.546 to -0.243)	<0.001

Spearman’s rank correlation was used, with FPN1–8C>G genotypes coded as CC=1, CG=2, and GG=3. Positive and negative ρ values indicate increasing and decreasing biomarker levels across genotypes, respectively. 95% CIs were estimated by non-parametric bootstrapping. p<0.05 was considered significant. TIBC = total iron-binding capacity; UIBC = unsaturated iron-binding capacity; ERFE = erythroferrone; GDF-15 = growth differentiation factor-15; sTfR = soluble transferrin receptor; CI = confidence interval.

## DISCUSSION

Despite advances in transfusion support and iron chelation, iron overload remains a major cause of morbidity in transfusion-dependent β-thalassemia major.^21^ In the present study, the FPN1–8C>G polymorphism was significantly associated with iron overload, and this association remained significant after adjustment for iron-chelation regimen and duration of transfusion exposure. These findings suggest that *SLC40A1* variation may contribute to inter-individual differences in iron burden; however, the cross-sectional design precludes inference of a causal relationship.

Our findings partly support El-Gharbawi et al., who reported an association between FPN1–8C>G and increased liver iron concentration in Egyptian patients with β-thalassemia major.^10^ In contrast to their study, which found no significant association with serum ferritin, the present cohort demonstrated progressively higher serum iron, ferritin, and transferrin saturation across CC, CG, and GG genotypes. Differences in population characteristics, treatment exposure, and methods used to assess iron burden may explain these variations. The present study therefore extends the limited evidence on FPN1–8C>G to a Pakistani population.

The genotype-associated biomarker profile also indicated altered iron regulation. Increasing variant dosage was associated with higher ERFE and GDF-15 levels and lower hepcidin levels, consistent with dysregulation of the erythroid–hepcidin axis in β-thalassemia.^13^,^22^ sTfR levels decreased across genotypes, as it primarily reflects erythropoietic activity rather than body iron stores. This finding may reflect suppression of endogenous erythropoiesis in regularly transfused patients.^23^

Although all participants were receiving HbF augmentation therapy, the study was not designed to evaluate a genotype–hydroxyurea interaction; therefore, these findings should not be interpreted as evidence that FPN1–8C>G modifies therapeutic response.

### Limitations

The cross-sectional design limited assessment of causal and temporal relationships. Residual confounding from transfusion and chelation-related factors may remain despite statistical adjustment. In addition, organ-specific iron burden was not assessed by MRI, and the small number of patients with the GG genotype may have reduced the precision of genotype-specific estimates.

## CONCLUSION

The FPN1–8C>G polymorphism was significantly associated with greater iron burden and altered iron-regulatory biomarkers in transfusion-dependent β-thalassemia major patients receiving HbF augmentation therapy. The association remained significant after adjustment for iron-chelation regimen and duration of transfusion exposure, suggesting a potential contribution of FPN1–8C>G to inter-individual variability in iron overload. However, the cross-sectional design does not establish causality.

### Recommendations

Further prospective, multicenter studies with larger cohorts and MRI-based assessment of hepatic and cardiac iron are required to validate FPN1–8C>G as a potential marker of iron-overload susceptibility and to determine its prognostic and clinical utility before routine genotypic screening is considered.

### Author Contributions:

**MW:** Contributed to the conceptualization and design of the study, data collection, laboratory work, statistical analysis, data interpretation, manuscript drafting

**MYJS:** Contributed to study supervision, methodological design, data interpretation, critical revision of the manuscript.

**IN:** Contributed to patient recruitment, data collection, study coordination, data interpretation, manuscript review,

**MR:** Contributed to institutional oversight, clinical coordination, critical review and editing of the manuscript.

All authors have read and approved the final manuscript and agree to be accountable for all aspects of the work.

## References

[1] Sohail G, Taj AS, Naz A, Khan MTM (2025). Genetic profiling and frequencies of modifiers in transfusion-dependent thalassemia. Pak J Med Sci.

[2] Ejaz S, Abdullah I, Usman M, Iqbal MA, Munawar S, Khan MI (2023). Mutational analysis of hemoglobin genes and functional characterization of detected variants through in-silico analysis in Pakistani beta-thalassemia major patients. Sci Rep.

[3] Faruqi A, Zafar T, Subuctageen S, Mughal IA (2024). Iron overload and liver function in patients with beta thalassemia major: a cross-sectional study. Pak J Med Sci.

[4] Basu S, Rahaman M, Dolai TK, Shukla PC, Chakravorty N (2023). Understanding the intricacies of iron overload associated with β-thalassemia: a comprehensive review. Thalass Rep.

[5] Sadiq IZ, Abubakar FS, Usman HS, Abdullahi AD, Ibrahim B, Kastayal BS (2024). Thalassemia: pathophysiology, diagnosis, and advances in treatment. Thalass Rep.

[6] Camaschella C, Nai A, Silvestri L (2020). Iron metabolism and iron disorders revisited in the hepcidin era. Haematologica.

[7] Uguen K, Ka C, Collod-Béroud G, Le Tertre M, Guellec J, Férec C (2023). The spectra of disease-causing mutations in the ferroportin 1 (*SLC40A1*) encoding gene and related iron overload phenotypes (hemochromatosis type 4 and ferroportin disease). Hum Mutat.

[8] Nemeth E, Ganz T (2021). Hepcidin-ferroportin interaction controls systemic iron homeostasis. Int J Mol Sci.

[9] Uguen K, Le Tertre M, Tchernitchko D, Elbahnsi A, Maestri S, Gourlaouen I (2024). The dual loss and gain of function of the FPN1 iron exporter results in the ferroportin disease phenotype. HGG Adv.

[10] El-Gharbawi N, Shaheen I, Hamdy M, Elgawhary S, Samir M, Hanna BM (2023). Genetic variations of ferroportin-1 (FPN1-8CG), TMPRSS6 (rs855791) and hemojuvelin (I222N and G320V) among a cohort of Egyptian β-thalassemia major patients. Indian J Hematol Blood Transfus.

[11] Dissanayake R, Samarasinghe N, Waidyanatha S, Pathirana S, Neththikumara N, Dissanayake VHW (2022). Assessment of iron overload in a cohort of Sri Lankan patients with transfusion-dependent beta-thalassaemia and its correlation with pathogenic variants in *HBB, HFE, SLC40A1,* and *TFR2* genes. BMC Pediatr.

[12] Prahasanti K, Ashariati A, Herawati L, Romadhon PZ, Mahdi BA, Dzakiyah AZ (2026). Ineffective erythropoiesis markers in β-thalassemia: a systematic review. J Clin Med.

[13] Youssry I, Samy RM, AbdelMohsen M, Salama NM (2024). The association between growth differentiation factor-15, erythroferrone, and iron status in thalassemic patients. Pediatr Res.

[14] Meena S, Sharma K, Sharma S, Chandra J (2023). Study of growth differentiation factor-15 in polytransfused children with β-thalassemia. Indian J Pathol Microbiol.

[15] Salama M, Hassab H, Balbaa N, Ettaby A, Saad N, Hassanein N (2026). Serum erythroferrone level and its relation to iron overload in patients with beta-thalassemia. J Appl Hematol.

[16] Yasara N, Wickramarathne N, Mettananda C, Silva I, Hameed N, Attanayaka K (2022). A randomised double-blind placebo-controlled clinical trial of oral hydroxyurea for transfusion-dependent β-thalassaemia. Sci Rep.

[17] Huang T, Jiang H, Tang G, Li J, Huang X, Huang Z (2025). Efficacy and safety of hydroxyurea therapy on patients with β-thalassemia: a systematic review and meta-analysis. Front Med (Lausanne).

[18] Ansari SH, Shamsi TS, Ashraf M, Perveen K, Farzana T, Bohray M (2011). Efficacy of hydroxyurea in providing transfusion independence in β-thalassemia. J Pediatr Hematol Oncol.

[19] Farmakis D, Porter J, Taher A, Cappellini MD, Angastiniotis M, Eleftheriou A (2022). 2021 Thalassaemia International Federation guidelines for the management of transfusion-dependent thalassemia. HemaSphere.

[20] Taher AT, Saliba AN (2017). Iron overload in thalassemia: different organs at different rates. Hematology Am Soc Hematol Educ Program.

[21] Daar S, Al Khabori M, Al Rahbi S, Hassan M, El Tigani AB, Pennell DJ (2020). Cardiac T2* MR in patients with thalassemia major: a 10-year long-term follow-up. Ann Hematol.

[22] Babar S, Siddiqi MYJ, Rizwan M, Ahmed S, Saboor M (2026). Comparative analysis of erythroferrone and hepcidin as emerging biomarkers of iron homeostasis in patients with β-thalassemia major: a case-control study from Pakistan. Hemoglobin.

[23] Danise P, Amendola G, Di Concilio R, Cillari E, Gioia M, Di Palma A (2009). Nucleated red blood cells and soluble transferrin receptor in thalassemia syndromes: relationship with global and ineffective erythropoiesis. Clin Chem Lab Med.

